# Vestibular function in carotid territory stroke patients

**DOI:** 10.5935/1808-8694.20130005

**Published:** 2015-10-14

**Authors:** Anna Paula Batista de Ávila Pires, Marcia Maiumi Fukujima, Fernando Freitas Ganança, Letícia de Moraes Aquino, Maurício Malavasi Ganança, Heloisa Helena Caovilla

**Affiliations:** MSc in Sciences by the Paulista Medical School of the University of São Paulo (ENT); PhD in Sciences (neurology) by the Paulista Medical School of the Federal University of São Paulo (Neurologist); PhD in Medicine (ENT) by the Paulista Medical School of the Federal University of São Paulo (Adjunct Professor in the Otology and Otoneurology program) (ENT); MSc in Sciences by the Paulista Medical School of the University of São Paulo (Physical Therapist); PhD in Medicine (ENT) by the Paulista Medical School of the Federal University of São Paulo (Professor of Otorhinolaryngology) (ENT); PhD in Human Communication Disorders by the Paulista Medical School of the Federal University of São Paulo (Associate Professor of Otoneurology) (Speech and Hearing Therapist). Otology and Otoneurology program

**Keywords:** dizziness, electronystagmography, stroke, vertigo

## Abstract

Stroke patients may present otoneurological symptoms.

**Objective:**

To assess the vestibular function of subjects with a history of carotid territory stroke.

**Method:**

This historical cohort cross-sectional study enrolled 40 patients; subjects answered the Dizziness Handicap Inventory, were interviewed and submitted to ENT examination and vector electronystagmography.

**Results:**

Mild saccadic movement anomalies were seen in 20 patients (50.0%); nine complained of imbalance and dizziness. Abnormal smooth pursuit gain was seen in 17 cases (42.5%); six subjects reported imbalance and one complained of dizziness. Abnormal directional preponderance during rotational nystagmus was seen in two cases (5.0%), who also reported imbalance. Three patients (7.5%) and two subjects (5.0%) were found to have abnormal labyrinthine predominance and abnormal nystagmus directional preponderance respectively; all five individuals reported imbalance. Ten of the 11 patients without complaints of disordered balance had altered saccadic and smooth pursuit eye movements, while one had unaltered vestibular function.

**Conclusion:**

Patients with a history of carotid territory stroke may suffer from dizziness or imbalance and present signs of compromised eye motility and vestibular function.

## INTRODUCTION

Stroke may be defined as an abrupt involvement of the cerebral vascular system that generates neurological deficit, varying degrees of incapacitation, and often death. Approximately 85% of strokes are ischemic in nature, and 15% are the consequence of brain hemorrhage. Stroke is one of the most significant public health issues because of its significant impact upon society and high cost of treatment and rehabilitation, in addition to frequent patient incapacitation. It is estimated that 0.5 to one case of stroke occurs for every 1000 people per day^1^. In Brazil, approximately 20% of the diagnosed neurological diseases are defined as stroke. The estimated incidence of ischemic stroke ranges between 0.6 and one case for every 1000 people per day^2^.

Sequelae after stroke vary depending on the topography, type, and extension of the lesion, but revolve mostly around cognitive, motor, sensory, and autonomic impairment^1^.

Vestibular function tests may be used to assess postural stability through the vestibulospinal reflex in static and dynamic balance studies and posturography, and the vestibulo-ocular interactions through the vestibulo-ocular reflex (VOR) in electronystagmography (ENG), vector electronystagmography (VENG) or videonystagmography (VNG)^3^^,^^4^.

VENG is a type of ENG that utilizes three channels to record eye movements. It helps understand the status of vestibular function and recognize the side and site of lesions in cases of vestibular system dysfunction^3^^,^^4^.

Little information is available on the vestibular function assessment of patients with a history of stroke, as most studies focus on the acute stage of the disease^5^, ^6^, ^7^, ^8^, ^9^, ^10^, ^11^, ^12^. The assessment of long-term stroke patients may help describe the vestibulo-ocular involvement and assist in the production of topodiagnostic, prognostic, and therapeutical advice over the possible balance disorders directly or indirectly related to stroke and its causes.

This study aimed to assess the vestibular function of patients with a history of carotid territory stroke.

## METHOD

This cross-sectional descriptive analytical quantitative study was approved by the Research Ethics Committee of the institution (permit 0989/09).

Forty long-term carotid territory stroke patients were enrolled (n = 40, in accordance with the central limit theorem) in the study. Their status was confirmed by clinical examination and/or imaging tests (skull CT or MRI scans); patients had to be able to walk and maintain their bodies in an erect standing position.

Exclusion criteria included: vertebrobasilar involvement; pre-stroke clinical diagnosis or symptoms of vestibular disorder; hemodynamic alterations (heart rate or blood pressure) contraindicating body balance assessment; other associated or previous diseases resulting in bone and muscle sequelae that prevented the subjects from being tested; inability to understand and follow simple verbal commands and scores below 24 in cognitive testing - mini-mental state examination (MMSE)^13^; inability to independently maintain the body in an erect standing position; use of gait assistive devices; severe visual involvement or involvement not compensated by the use of corrective lenses; orthopedic disorders resulting in movement limitation and use of lower limb prosthetics; psychiatric disorders; reported alcohol intake within 24 hours prior to assessment or medical diagnosis of chronic alcohol abuse; use of medications that affect the central nervous or the vestibular system (psychotropic drugs); body balance rehabilitation sessions within the last six months.

The patients were asked to answer a questionnaire and provide data on their social and demographic status, neurological and otoneurological disorders, functional involvement, habits, quality of life, body balance, and occurrence of falls. They also underwent clinical ENT examination and had their outer ear canals visually inspected. The Brazilian version of the Dizziness Handicap Inventory (DHI)^14^^,^^15^ was applied and vestibular function was asses-sed^3^^,^^4^^,^^16^. Data on the acute stages of stroke was gathered from the patients' charts.

Data on neurological, otoneurological, and functional involvement was based on medical and topographic diagnosis, type of stroke, personal history, number and type of drugs in use, physical therapist's diagnosis of the subjects' motor and sensorial status, status of dizziness and other symptoms correlated with possible post-stroke vestibular dysfunction. In particular, patients were asked about how often they had dizzy spells, the type of spells they had, their duration, intensity, and triggering positions or tasks, during and since they had the stroke. Subjects were asked about associated otoneurological symptoms, such as tinnitus, aural fullness, hypersensitivity to sounds, hearing loss, oscillopsia, headache, sweating/paleness/ tachycardia, nausea, vomiting, insomnia, fear, feeling of passing out, memory and concentration disorders.

The DHI^14^^,^^15^ is made up of 25 questions. Questions 01; 04; 08; 11; 13; 17 and 25 assess the physical domain; questions 02; 09; 10; 15; 18; 20; 21; 22 and 23 look into the emotional domain; and questions 03; 05; 06; 07; 12; 14; 16; 19 and 24 investigate the functional domain. Possible answers are ‘yes,' ‘no,' or ‘sometimes.' Each affirmative answer adds four points to the subject's score. Negative answers score no points, while ‘sometimes,' add two points to the patient's score. The specific scores of each domain were computed along with the total score, and the mean scores of the different domains were considered. The impact upon quality of life is considered mild for scores ranging between 0 and 30, moderate in the 31-60 range, and severe when scores reach the 61-100 interval^17^. The highest possible score is 100, indicating maximum harm caused by dizziness, while the lowest is zero - or no harm done by dizziness.

Vestibular function assessment^3^, ^4^^,^^16^ included static and dynamic balance tests with open and closed eyes (Romberg, Romberg-Barré, Unterberger-Fukuda, and gait) tests for positional and positioning nystagmus, spontaneous, fixation, and optokinetic nystagmus, fixed and randomized saccadic eye movements, smooth pursuit, tracking tests, and caloric test with air at 50°C and 24°C during VENG (VECWIN device, light bar and air caloric stimulator, *Neurograff Eletromedicina Ind. e Com. Ltda. - EPT)*. Before undergoing VENG^3^, patients were instructed to refrain from taking medication for dizziness and psychotropic drugs for 72 hours, and not to drink tea, coffee, soda, or alcohol or to eat chocolate or smoke for 48 hours, to avoid interfering with eye movements and thus altering test results. On the day of the tests they were advised to have light meals and to fast for three hours before taking the tests.

In order to assess vertigo and positioning nystagmus, patients were initially placed in a seated position with their heads tilted 45 degrees to one side, and were then led quickly into opposite-side lateral decubitus; then they were seated again and the procedure was repeated to the other side. The subjects were kept on each of the positions for 30 seconds or until dizziness and/or nystagmus diminished or disappeared completely. Before the test, patients were instructed not to resist the maneuver and not to close their eyes. In the presence of nystagmus, in addition to concomitant vertigo, direction, duration, paroxysm, and fatigability were considered.

In vertigo and positional nystagmus assessment the subjects were aided by the examiner and moved slowly from a seated to a supine position, turning their heads to the right and then going back to a supine position. The maneuver was repeated with the patients turning their heads to the left. Then patients moved to right lateral decubitus, supine position, and left lateral decubitus before moving back again to a seated position. The subjects were kept on each of the positions for 30 seconds. In the presence of nystagmus, in addition to concomitant vertigo, direction, duration, paroxysm, and fatigability were considered.

During VENG^3^^,^^4^, one ground and three active electrodes were placed in the subjects' periorbital areas after the skin in the region had been cleaned. The active electrodes were placed in the outer corner of the right periorbital area, in the outer corner of the left periorbital area, and in the frontal midline in a triangular arrangement, which allowed eye movements to be recorded in three channels.

Calibration was performed so that the various stages of the test could be carried out in the same conditions and to allow automatic measurement of latency, accuracy, speed, and gain of other eye movements, in addition to the slow component of nystagmus.

The presence of spontaneous nystagmus was analyzed in frontal gaze, first with open and then closed eyes. Fixation nystagmus was investigated with the patients gazing to the right, left, up, and down, without going over 30° away from the midline. The occurrence of these phenomena and their intensities were assessed through the slow component speed calculated automatically.

Saccadic movements were assessed with the subjects following a target moving on fixed and random patterns; considered parameters included latency, speed, and saccade accuracy. In the smooth pursuit tests the patients were asked to use their eyes to follow a source of light moving on a sinusoidal pattern at 0.1, 0.2, and 0.4 Hz; ocular movement type and gain were analyzed. Optokinetic nystagmus speed and gain were measured while the subjects followed a source of light moving first in one and then in another direction at a speed of 40°/s. Alterations in saccadic movement, smooth pursuit, and optokinetic nystagmus were determined in comparison with reference values^18^ included in the testing equipment.

Tracking tests were carried out with the patients in a seated position with eyes closed and heads tilted forward by 30° in order to stimulate the lateral semicircular canals. In order to stimulate the posterior and superior semicircular canals, the subjects were asked to tilt their heads backwards by 60° and to the right by 45° degrees, and then backwards by 60° and to the left by 45°. The seat was shifted by 90° from its central position and released, producing a pendular movement of decreasing amplitude. Nystagmus triggered during the test was assessed through the measurement of its slow component. Presence of pre-test nystagmus and its possible impacts on the outcomes of the test were also considered. Directional preponderance values of 25.0% and under for lateral semicircular canal, 27.0% for posterior semicircular canal and 26.0% for anterior semicircular canal stimulation were considered to be within normal range^18^.

Caloric air tests^3^^,^^4^^,^^16^ were carried out by stimulating each ear separately with air at 50°C and 24°C for 60 seconds, in intervals of three minutes between sessions. Presence of pre-caloric test nystagmus and its possible impact on test results were investigated. Vertigo, direction, and speed of the slow component in post-caloric test nystagmus were analyzed for patients with open and closed eyes. Labyrinthine and directional preponderance values were calculated. Labyrinthine preponderance values of 25.0% and under and directional preponderance values of up to 30.0% were deemed normal^19^.

The identification of vestibular function involvement and its location in the peripheral (labyrinth and/or vestibular nerve) or central nervous system structures was based on the subjects' clinical history and otoneurological findings^3^^,^^4^.

The patients were characterized in terms of their age, gender, mean time since the occurrence of stroke, stroke topography as confirmed by imaging tests, type of dizziness during the occurrence of stroke and at the time of the study, ENT and vestibular function examination findings, presence or absence of alterations in dynamic and static balance tests, positional and positioning nystagmus, spontaneous, fixation, and optokinetic nystagmus, saccadic eye movements, smooth pursuit, tracking tests, and caloric tests. Vestibular function was considered abnormal when alterations were seen in at least one of the tests mentioned above. Complaints of dizziness and imbalance at the time the study was carried out were compared to normal or altered vestibular function test results.

Descriptive statistical analysis was conducted to characterize the sample, considering the data found to be relevant for the purposes of this study. Quantitative variables were expressed in the form of mean values ± standard deviations when under a normal distribution, and in the form of median, minimum, and maximum values when not under a normal distribution. Qualitative variables were presented in the form of absolute and relative frequencies. Fisher's exact test was used to analyze ratio homogeneity. A significance level of 5% was adopted.

## RESULTS

Forty patients aged between 44 and 83 years (median 63.53; 44-83 years) were enrolled in the study; twenty-two (55.0%) were females and 18 were males. The mean time between stroke and enrollment in the study was 65.2 months. In terms of stroke topography, 22 patients (55.0%) had left middle cerebral artery (MCA) involvement, 16 (40.0%) had right MCA involvement, one (2.5%) had left anterior cerebral artery (ACA) involvement, and one (2.5%) had right ACA involvement. All patients were diagnosed with ischemic carotid territory stroke, further confirmed by imaging.

Table 1 shows the prevalence rates of types of dizziness at the time of stroke and at the time the study was carried out.

**Table 1:** Prevalence rates of dizziness types of 40 patients at the time of carotid territory stroke and in long-term follow-up at the time of this study.

	Dizziness type		Acute Stage N %	Long-term follow-up N %
Non-rotational dizziness	21	52.5	3	7.5
Vertigo	9	22.5	0	0.0
Imbalance	5	12.5	26	65.0
No complaints of body imbalance	3	7.5	11	27.5
Vertigo and imbalance	2	5.0	0	0.0

Table 2 describes the mean values and standard deviations, and the median, minimum, and maximum values of total scores and scores in the physical, emotional, and functional domains of the DHI of 40 carotid territory stroke patients.

**Table 2:** Dizziness Handicap Inventory scores of 40 long-term carotid territory stroke patients.

Dizziness Handicap Inventory	Mean	SD	Median	Minimum	Maximum
Functional Domain	6.75	5.45	6	0	24
Physical Domain	6.25	4.86	6	0	24
Emotional Domain	6.40	6.13	5	0	24
Total Score	19.40	7.15	9	0	64

All 40 long-term stroke patients had normal ENT examination findings.

Table 3 draws a comparison between complaints of dizziness and imbalance at the time of the study and the alterations identified in vestibular function assessment; no statistically significant differences were noted between the reports of dizziness and imbalance and alterations in vestibular function (*p* = 0.276). Thirteen (32.5%) patients had normal results in the tests used to assess vestibular function; ten of them (25.0%) reported imbalance, two (5.0%) complained of dizzy spells, and one (2.5%) had no complaints of dizziness or imbalance. Twenty-seven (67.5%) patients had abnormal vestibular function parameters; 16 of them (40.0%) reported imbalance, one (2.5%) complained of dizziness, and ten (25.0%) had no complaints of dizzy spells or imbalance episodes. None of the 40 patients had altered test results in static and dynamic balance tests with open or closed eyes (Romberg, Romberg-Barré, Unterberger-Fukuda and gait), positioning and positional nystagmus tests, or in spontaneous (open or closed eyes), fixation, and optokinetic nystagmus tests.

**Table 3:** Comparison between complaints of dizziness and imbalance and presence of vestibular alterations of 40 long-term carotid territory stroke patients.

Vestibular examination	With complaints of dizziness/ imbalance	Without complaints of dizziness/ imbalance	Total	*p*-value*
Normal	12	1	13	
Abnormal	17	10	27	276
Total	29	11	40	

SD: Standard Deviation.

* Fisher's exact test.

Mild abnormalities in latency, accuracy, and/or speed of fixed/random saccadic eye movements with preserved morphology were seen in 20 patients (50.0%); nine patients referred imbalance and one reported dizziness at the time of the study.

Smooth pursuit was abnormal for gain values in 17 cases (42.5%), six of which complained of imbalance and one of dizziness at the time of the study.

Nystagmus during tracking tests revealed abnormal directional preponderance in one case (2.5%) when lateral, anterior, and posterior semicircular canals were stimulated, and in one case (2.5%) during posterior and anterior canal stimulation; both reported imbalance at the time of the study.

Caloric air tests found three patients (7.5%) with abnormal labyrinthine preponderance and two (5.0%) with abnormal nystagmus directional preponderance; all five subjects reported imbalance at the time of the study.

Ten of the eleven patients who did not report altered body balance at the time of the study had altered fixed/random saccadic and smooth pursuit eye movements.

## DISCUSSION

This study looked into the vestibular function of 40 patients, four to eight years after they had had carotid territory stroke, by means of interviews, clinical ENT examination, the DHI^14^^,^^15^, and assessment of vestibular function through vector electronystagmography^3^^,^^4^. Vestibular function has been analyzed in acute stroke patients^5^, ^6^, ^7^, ^8^, ^9^, ^10^, ^11^, ^12^, while one study followed stroke patients for 14 to 85 months^7^.

There was significant prevalence in our series of stroke involving the middle and anterior cerebral arteries, i.e., the anterior or carotid circulation, in patients of both genders in their sixties. Other studies have agreed with the finding of stroke patients in their sixties and seventies, albeit with a slight predominance among males^1^^,^^2^. In acute stroke, most patients (92.5%) had non-rotational dizziness, vertigo, and/or imbalance; only three patients (7.5%) did not report one or more of these symptoms. Contrary to our findings, typical vertigo was not seen in acute supratentorial stroke and few patients reported non-rotational dizziness, although today the cortical projections of the vestibular nuclei have been considered as possible originators of vertigo secondary to cerebral hemisphere injury (supramarginal gyrus in the parietal lobe, insular cortex, posterior insula)^6^. It has been traditionally considered that brainstem and cerebellar central vestibular injuries may produce vertigo; the occurrence of vertigo in stroke patients has been debated^6^. Dizziness and imbalance were more prevalent in stroke patients with vertebrobasilar involvement, i.e., in the posterior vascular territory^8^, ^9^, ^10^, ^11^, ^12^.

Dizziness and/or imbalance were reported by most of our patients (92.5%) in the acute stages of stroke and also by the majority of the stroke patients (72.5%) in long-term follow-up - the time at which this study was carried out. These symptoms, whether or not in association, were also reported by patients at the time of stroke^6^^,^^9^^,^^11^^,^^12^. Long-term stroke patients have reported significant improvement from symptoms of imbalance, but most still complain of dizzy spells triggered by quick head movements^7^.

Long-term stroke patients had similar scores in the physical, functional, and emotional domains of the DHI; the impact of dizziness and other symptoms related to body balance upon quality-of-life was rated as mild^17^. No references were found as to the use of this questionnaire to assess carotid territory stroke patients.

VENG revealed mild alterations in the latency, speed, and/or accuracy of saccadic movements of the long-term stroke patients with preserved morphology enrolled in this study, and in gain and smooth pursuit in cases with or without disordered body balance. Saccadic movement alterations indicate impaired control of the central nervous system over rapid eye movements, while smooth pursuit alterations suggest oculomotor system involvement with disordered slow eye movement control^3^. No references were found on saccades and smooth pursuit in long-term carotid territory stroke patients. The identified alterations, although mild, may suggest central nervous system injury^20^. Acute stroke patients have been found to have altered smooth pursuit and/or saccadic movements^21^^,^^22^; bilateral horizontal fixation nystagmus, vertical spontaneous nystagmus, absent fixation suppression, saccadic dysmetria, and altered optokinetic nystagmus^11^, spontaneous nystagmus, anomalous slow pursuit and saccadic movements^5^; anomalous smooth pursuit and asymmetric optokinetic nystag-mus^10^; horizontal spontaneous nystagmus and altered eye tracking tests^11^; eye tracking alterations and bi-directional fixation nystagmus^12^.

The tracking tests performed in the long-term stroke patients enrolled in this study revealed directional preponderance in nystagmus during rotation in patients who reported imbalance; one subject presented this anomaly when his lateral and vertical semicircular canals were stimulated, while another had it during vertical semicircular canal stimulation. This finding indicates vestibular system decompensation^3^. No references were found on tracking tests of long-term carotid territory stroke patients.

Caloric tests elicited vestibular hypofunction or directional preponderance in five long-term stroke patients with imbalance. These signs are indicative of involved vestibular function^3^. Hyporeflexia, as identified at the time of stroke, persisted in 23.0% of the patients for at least a year, and has been found to disappear in subjects followed for five or more years^7^. Close to the time of stroke, patients were found to have nystagmus alterations after caloric testing such as hyperreflexia e hyporeflexia^9^, inversion^5^, areflexia and hyporeflexia^10^ and hypofunction^12^.

Dizziness and/or imbalance along with vestibular disorders were frequently seen in our long-term carotid territory stroke patients. However, there was no significant statistical correlation between abnormal VENG findings and these complaints, as in subjects without complaints the VENG findings were also abnormal in most instances, which could indicate the occurrence of asymptomatic sequelae in these subjects. Classic vestibular examination as performed in this study usually provides little information on the diagnosis of supratentorial injuries. However, in our series, the mild latency, accuracy, and/or speed alterations of fixed/random saccadic movements and abnormal smooth pursuit gain could stem from cerebral hemisphere involvement^3^^,^^23^^,^^24^.

Therefore, one cannot rule out a correlation between vestibular manifestations and history of stroke or its assumed causes. These symptoms and/or vestibulo-ocular alterations may correspond to sequelae from the vascular involvement of the central nervous system, arise from comorbidities commonly seen in the elderly or in earlier peripheral vestibular disorders related or not to the etiology of stroke, or to the use of one of more drugs to treat them. Long-term stroke patients with symptoms suggestive of vestibular dysfunction must be assessed from the otoneurological standpoint in order to be given proper therapeutic guidance when needed.

## CONCLUSION

Long-term carotid territory stroke patients may present dizziness or imbalance and signs of oculomotor and vestibular system disorders.
